# Depression and Anxiety Symptoms in Adults Displaced by Natural Disasters

**DOI:** 10.1001/jamanetworkopen.2025.28546

**Published:** 2025-08-22

**Authors:** Ther W. Aung, Kari O’Donnell, Susan De Luca, Douglas Gunzler

**Affiliations:** 1 Population Health and Equity Research Institute, The MetroHealth System, Cleveland, Ohio; 2 School of Medicine, Case Western Reserve University, Cleveland, Ohio

## Abstract

**Importance:**

Mental health symptoms associated with disaster-induced displacement on a national scale are unknown. Knowledge of mental health outcomes can inform disaster management and recovery.

**Objective:**

To investigate the association of depression and anxiety symptoms with disaster-induced displacement and the duration of displacement.

**Design, Setting, and Participants:**

This cross-sectional study analyzed data from the US Census Bureau Household Pulse Survey, which was administered nationwide online between December 9, 2022, and October 30, 2023. One adult per selected US household with an email and/or mobile telephone number was eligible to participate.

**Exposures:**

Natural disaster–induced displacement from home in the past 12 months and the duration of displacement.

**Main Outcomes and Measures:**

Depression and anxiety symptoms were assessed using shortened (2-item) versions of the Patient Health Questionnaire and the Generalized Anxiety Disorder questionnaire. The composite questionnaire scores were used to determine the participants’ symptoms.

**Results:**

The weighted sample included 183 730 553 US adults (mean [SD] age, 49.22 [16.65] years; 94 445 996 females at birth [51.4%]). In multivariable analyses, individuals who were displaced for more than a week had higher odds of depression (odds ratio [OR], 1.77; 95% CI, 1.40-2.25) and anxiety (OR, 1.54; 95% CI, 1.22-1.94) symptoms compared with those who were not displaced. The longer the duration of displacement, the higher the odds of reporting depression and anxiety symptoms; individuals who never returned home reported higher odds of depression (OR, 2.43; 95% CI, 1.70-3.49) and anxiety (OR, 3.04; 95% CI, 2.10-4.39) symptoms. Socially vulnerable populations, such as younger adults, sexual and gender minority groups, and people with lower educational level, lower annual income, and disabilities, had a higher prevalence of depression and anxiety symptoms.

**Conclusion and Relevance:**

This study found that, in US adults, disaster-induced displacement from home was associated with increased odds of depression and anxiety symptoms, with the highest odds among those who never returned home. There is an urgent need to address mental health care for populations displaced by natural disasters, particularly socially vulnerable populations.

## Key Points

**Question:**

How is displacement from home by natural disasters and the duration of the displacement associated with depression and anxiety symptoms among the US adult population?

**Findings:**

In a cross-sectional study of a weighted sample of 183 730 553 adults, natural disaster–induced displacement was associated with increased odds of reporting depression and anxiety symptoms. Individuals who did not return home in the past 12 months had the highest odds of mental health symptoms compared with those with shorter duration of displacement.

**Meaning:**

As natural disasters increase in scale and frequency, there is an urgent need to address mental health care among adults displaced by natural disasters.

## Teaser

This cross-sectional study examines self-reported mental health outcomes among a nationally representative sample of US adults who experienced disaster-induced displacement in the past 12 months.

## Introduction

Disaster-induced displacement, when individuals are forced to leave their homes due to natural or person-made disasters, has substantial implications for mental health.^1,2,3^ The psychological impact of such displacement is profound and multifaceted, often associated with increased rates of anxiety, depression, and posttraumatic stress disorder among affected populations.^4,5,6^ Individuals who experience displacement encounter a variety of stressors, including housing instability or losing their family home, unemployment or decreased financial stability, and lack of community resources, which can intensify feelings of isolation and hopelessness.^7^

Much of the literature on disaster-induced displacement and mental health outcomes is dominated by studies outside of the US,^2,8^ limiting the application of the findings to the US population. The few US-based studies that exist are limited by relatively small sample sizes, with many of them involving from 100 to a few hundred participants.^3,9,10,11^ Compounding this challenge is the lack of a standardized questionnaire that defines mental health symptoms, which limits the generalizability of the evidence.^6^ Another key knowledge gap is that studies of displaced residents have rarely included those who never returned home, as most studies survey only those who return to disaster sites.^12^ Additionally, previous studies have focused on a type of disaster, such as hurricanes,^9,13^ or individual disasters,^9,13^ thus limiting comparisons of mental health outcomes of different disasters and types of disasters across larger geographic regions of the US.

We used data from a previous US Census Bureau Household Pulse Survey (HPS), which was administered to adults nationwide and included questions on displacement by natural disasters, the duration of displacement, and mental health outcomes. This study’s objective was to investigate the association of depression and anxiety symptoms with disaster-induced displacement and the duration of displacement. As climate change is expected to increase the frequency and intensity of natural disasters,^14^ knowledge of the extent of outcomes associated with characteristics of displacement can inform mental health service models for disaster-displaced populations in the US.

## Methods

The study used data from the cross-sectional online HPS conducted between December 9, 2022, and October 30, 2023 (survey weeks 52-63). The sample was drawn from approximately 130 million housing units derived from the Census Bureau’s Master Address File. More than 85% of addresses in the Master Address File matched with a contact frame consisting of telephone numbers and/or email addresses and were eligible for the sample. One adult respondent from each of the selected households was invited via text and email to complete the survey online. In accordance with the Common Rule, this cross-sectional study was exempt from ethics review and informed consent requirement because deidentified data were used. We followed the Strengthening the Reporting of Observational Studies in Epidemiology (STROBE) reporting guideline.^15^

The HPS provides person weights that, when applied, are nationally representative of the US adult population. The Census Bureau applies an adjustment to the person weights, which considers nonresponse bias, the occupied housing unit ratio based on the 2021 American Community Survey, and the number of adults living in the sample household. Additionally, it uses an iterative raking procedure to adjust person weights to population controls from Census estimates (ie, the 2021 American Community Survey and the 2022 and 2023 Population Estimates Program) based on demographic characteristics, including age, sex, race, ethnicity, and educational level, within each state.^16^

### Outcomes

Depression and anxiety outcomes were assessed with a modified version of the 2-item Patient Health Questionnaire (PHQ-2) and the 2-item Generalized Anxiety Disorder (GAD-2), respectively.^17^ Both questionnaires asked about symptoms occurring in the past 2 weeks. For example, the PHQ-2 asked, “Over the last two weeks, how often have you been bothered by … (1) having little interest or pleasure in doing things and (2) feeling down, depressed, or hopeless?” The GAD-2 asked, “How often have you been bothered by … (1) feeling nervous, anxious, or on edge and (2) not being able to stop or control worrying?” Responses and scores included the following: 0 for not at all, 1 for several days, 2 for more than half the days, and 3 for nearly every day. The 2 responses for each scale were summed together, and a score equal to 3 or greater on PHQ-2 and GAD-2 was screened for a diagnosis of major depressive disorder and generalized anxiety disorder, respectively.^18,19^

### Exposures

The exposures of interest were natural disaster–displacement from home and the duration of the displacement. Beginning in December 2022, HPS respondents were asked, “In the past year, were you displaced from your home because of a natural disaster?” Those who responded yes were asked, “How long were you displaced from your home?” Responses included less than a week, more than a week but less than a month, 1 to 6 months, more than 6 months, and never returned to home. Those who responded no to the displacement question were not asked any questions about natural disasters, including disaster types. The no displacement group served as the reference group and may include populations who experienced natural disasters but were not displaced. Respondents with missing data on displacement were excluded from the analysis, as displacement is the primary exposure of interest. The eMethods in Supplement 1 provides details on sample selection.

### Covariates

We obtained self-reported sociodemographic characteristics, such as sex at birth, educational level, annual income, and marital status, from the HPS database. Continuous variables, such as age group, household size, and number of children in the home, were grouped into categorical data. For sexual orientation, the responses gay or lesbian, bisexual, something else, and I do not know were grouped into 1 category due to small sample sizes.

We assessed race and ethnicity in the study because an earlier analyses on correlates of disaster-induced displacement indicated that certain population groups, such as non-Hispanic Black and non-Hispanic other, were more likely to be displaced compared with non-Hispanic White.^20^ Race and ethnicity were self-reported and obtained from 2 separate questions. The HPS database listed 4 categories for race: Asian, Black, White, and other race or combination of races. The other category included American Indian or Alaska Native, Chamorro, Native Hawaiian, Other Pacific Islander, Samoan, or multiracial. Data on Hispanic ethnicity were reported in 2 categories: no—not of Hispanic, Latino, or Spanish origin; yes—of Hispanic, Latino, or Spanish origin. We combined the race and ethnicity variables following a Census practice^21^ and created 5 categories: Hispanic of any race, non-Hispanic Asian, non-Hispanic Black, non-Hispanic White, and non-Hispanic other (hereafter as Hispanic, Asian, Black, White, and other, respectively).

Functional disability was derived from a set of 6 questions on difficulty with the following: seeing, hearing, remembering or concentrating, walking or climbing stairs, self-care, and communicating. The responses included no—no difficulty, yes—some difficulty, yes—a lot of difficulty, and cannot do at all. We categorized the last 2 responses (yes—a lot of difficulty and cannot do at all) as having a disability.

### Statistical Analysis

Data were pooled across 12 independent survey weeks with a mean (SD) response rate of 6.6% (0.005%) and a mean (SD) number of respondents of 68 912 (5440) per survey week. The final study size was obtained after missing data, ranging from 0% (no missing data) to 7.7% for each variable (eTable in Supplement 1), were dropped from the study sample using listwise deletion. We used person weights provided by the Census Bureau to create nationally representative estimates for all of the analyses.

We generated descriptive statistics using weighted frequency and proportion. We assessed the bivariate association between outcomes (depression and anxiety symptoms) with each independent variable individually using the Rao–Scott χ^2^ statistics. We ran 2 multivariable logistic regression models to evaluate the association between depression and anxiety symptoms and disaster-induced displacement. The models were adjusted for sociodemographic characteristics to reduce confounding bias; covariates were all categorical data and included age group, sex at birth, sexual orientation, race and ethnicity, educational level, annual income, marital status, household size, number of children in the home, housing type, homeownership, functional disability, and Census region. We reported the odds ratio (OR) and 95% CIs for each independent variable.

Two-sided *P* < .05 was used to indicate statistical significance. We used R, version 4.4.1 (R Project for Statistical Computing), for all analyses and the survey package svyglm function (Thomas Lumley)^22^ for the weighted logistic regression. Data analyses were conducted from October 2024 to January 2025.

## Results

Of the 183 730 553 US adults in the sample, 2 429 909 (1.3%) reported natural disaster–induced displacement in the past year. The study population had a mean (SD) age of 49.22 (16.65) years, with 48 629 213 people (26.5%) in the 35 to 49 years age category, included 94 445 996 females at birth (51.4%) and 89 284 557 males at birth (48.6%); and 5.1% who self-identified as Asian, 10.0% as Black, 15.7% as Hispanic, 65.0% as White, and 4.2% as other race and ethnicity (Table 1). The majority had no children in the home (64.0%) and lived in a detached single-family home (68.2%).

**Table 1.** Study Population Characteristics

Characteristic	Full weighted sample, No. (%) (N = 183 730 553)
Displacement	
No displacement	181 300 644 (98.7)
<1 wk	1 055 617 (0.6)
>1 wk but <1 mo	603 114 (0.3)
1-6 mo	257 628 (0.1)
>6 mo	176 121 (0.1)
Never returned home	337 428 (0.2)
Age group, y	
18-25	12 910 601 (7.0)
26-34	32 114 374 (17.5)
35-49	48 629 213 (26.5)
50-64	47 798 068 (26.0)
65-74	31 203 102 (17.0)
≥75	11 075 195 (6.0)
Sex at birth	
Female	94 445 996 (51.4)
Male	89 284 557 (48.6)
Sexual orientation	
Straight	161 714 215 (88.0)
Gay, lesbian, or bisexual	22 016 338 (12.0)
Race and ethnicity^a^	
Hispanic	28 764 392 (15.7)
Non-Hispanic Asian	9 328 700 (5.1)
Non-Hispanic Black	18 451 519 (10.0)
Non-Hispanic White	119 508 811 (65.0)
Non-Hispanic other^b^	7 677 130 (4.2)
Educational level	
≤High school diploma	62 510 226 (34.0)
Some college or associate’s degree	54 383 077 (29.6)
≥Bachelor’s or graduate degree	66 837 250 (36.4)
Annual income, $	
<35 000	41 566 748 (22.6)
35 000-74 999	54 036 791 (29.4)
75 000-149 999	55 178 714 (30.0)
≥150 000	32 948 300 (17.9)
Marital status	
Now married	105 558 249 (57.5)
Widowed, divorced, or separated	33 998 377 (18.5)
Never married	44 173 927 (24.0)
Household size, persons	
1-2	80 149 144 (43.6)
3-4	69 667 758 (37.9)
≥5	33 913 651 (18.5)
No. of children in the home	
No children	117 614 597 (64.0)
1-2	52 263 036 (28.4)
≥3	13 852 921 (7.5)
Housing type	
Detached single-family	125 242 797 (68.2)
Attached single-family	13 447 003 (7.3)
Apartment	34 605 078 (18.8)
Other^c^	10 435 675 (5.7)
Homeownership	
Owner	129 341 389 (70.4)
Renter	54 389 164 (29.6)
Functional disability, severe	
No disability	154 515 378 (84.1)
1	21 855 330 (11.9)
≥2	7 359 845 (4.0)
Census region	
Northeast	31 034 205 (16.9)
South	69 897 331 (38.0)
Midwest	38 621 295 (21.0)
West	44 177 721 (24.0)

^a^ Self-reported by participants.

^b^ Includes American Indian or Alaska Native, Chamorro, Native Hawaiian, Other Pacific Islander, Samoan, and multiracial.

^c^ Includes mobile home, boat, RV (recreational vehicle), and van.

In the bivariate analyses, displacement was associated with a higher prevalence of depression and anxiety symptoms compared with nondisplacement. For example, depression symptoms were reported in 44.2% of participants who were displaced for more than 6 months compared with 21.6% in the nondisplaced population (Table 2). The prevalence of depression and anxiety symptoms was also highest among those in the youngest (18-25 years) compared with older age groups; who identified as gay, lesbian, or bisexual compared with straight; or with other race and ethnicity compared with Asian, Black, Hispanic, and White groups.

**Table 2.** Bivariate Correlates of Depression and Anxiety Symptoms

Characteristic	Patients, No. (%)	
	Depression symptoms (n = 40 056 618)	Anxiety symptoms (n = 51 473 559)
Displacement		
No displacement	39 171 782 (21.6)	50 435 300 (27.8)
<1 wk	279 972 (26.5)	349 172 (33.1)
>1 wk but <1 mo	238 783 (39.6)	261 879 (43.4)
1-6 mo	91 258 (35.4)	113 335 (44.0)
>6 mo	77 796 (44.2)	78 971 (44.8)
Never returned home	197 028 (58.4)	234 902 (69.6)
Age group, y		
18-25	4 691 372 (36.3)	5 580 393 (43.2)
26-34	9 582 503 (29.8)	12 191 218 (38.0)
35-49	11 048 577 (22.7)	14 934 048 (30.7)
50-64	9 305 518 (19.5)	11 995 656 (25.1)
65-74	4 193 385 (13.4)	5 241 105 (16.8)
≥75	1 235 264 (11.2)	1 531 140 (13.8)
Sex at birth		
Female	21 459 503 (22.7)	29 861 075 (31.6)
Male	18 597 115 (20.8)	21 612 484 (24.2)
Sexual orientation		
Straight	31 538 242 (19.5)	40 917 609 (25.3)
Gay, lesbian, or bisexual	8 518 376 (38.7)	10 555 951 (47.9)
Race and ethnicity^a^		
Hispanic	7 290 879 (25.3)	8 874 890 (30.9)
Non-Hispanic Asian	1 519 674 (16.3)	1 785 565 (19.1)
Non-Hispanic Black	4 212 471 (22.8)	5 033 484 (27.3)
Non-Hispanic White	24 835 421 (20.8)	32 990 016 (27.6)
Non-Hispanic other^b^	2 198 174 (28.6)	2 789 604 (36.3)
Educational level		
≤High school diploma	16 549 799 (26.5)	19 250 297 (30.8)
Some college or associate’s degree	14 007 250 (25.8)	17 743 068 (32.6)
≥Bachelor’s or graduate degree	9 499 570 (14.2)	14 480 195 (21.7)
Annual income, $		
<35 000	13 986 608 (33.6)	16 394 020 (39.4)
35 000-74 999	13 302 286 (24.6)	16 658 593 (30.8)
75 000-149 999	9 546 993 (17.3)	13 180 151 (23.9)
≥150 000	3 220 731 (9.8)	5 240 796 (15.9)
Marital status		
Now married	17 325 415 (16.4)	24 097 971 (22.8)
Widowed, divorced, or separated	8 571 864 (25.2)	10 456 935 (30.8)
Never married	14 159 339 (32.1)	16 918 653 (38.3)
Household size, persons		
1-2	16 038 845 (20.0)	20 351 425 (25.4)
3-4	15 459 740 (22.2)	20 260 560 (29.1)
≥5	8 558 033 (25.2)	10 861 574 (32.0)
No. of children in the home		
No children	25 379 614 (21.6)	31 713 763 (27.0)
1-2	11 378 087 (21.8)	15 267 045 (29.2)
≥3	3 298 918 (23.8)	4 492 752 (32.4)
Housing type		
Detached single-family	24 013 440 (19.2)	31 563 808 (25.2)
Attached single-family	2 920 085 (21.7)	3 785 360 (28.2)
Apartment	9 900 172 (28.6)	12 342 764 (35.7)
Other^c^	3 222 921 (30.9)	3 781 628 (36.2)
Homeownership		
Owner	23 066 122 (17.8)	30 593 908 (23.7)
Renter	16 990 497 (31.2)	20 879 651 (38.4)
Functional disability, severe		
No disability	25 904 871 (16.8)	35 505 449 (23.0)
1	9 396 896 (43.0)	10 913 881 (49.9)
≥2	4 754 852 (64.6)	5 054 229 (68.7)
Census region		
Northeast	6 183 140 (19.9)	8 209 048 (26.5)
South	16 202 779 (23.2)	20 409 330 (29.2)
Midwest	8 021 125 (20.8)	10 437 796 (27.0)
West	9 649 574 (21.8)	12 417 385 (28.1)

^a^ Self-reported by participants.

^b^ Includes American Indian or Alaska Native, Chamorro, Native Hawaiian, Other Pacific Islander, Samoan, and multiracial.

^c^ Includes mobile home, boat, RV (recreational vehicle), and van.

In our multivariable regression analyses, displacement was associated with depression and anxiety symptoms, particularly for participants who reported displacement of 1 week or longer (Table 3). For example, those who were displaced for more than 1 week but less than 1 month had higher odds of experiencing depression symptoms (OR, 1.77; 95% CI, 1.40-2.25) and anxiety symptoms (OR, 1.54; 95% CI, 1.22-1.94) compared with those who did not experience any displacement. In general, the longer the duration of displacement, the higher the odds of experiencing depression and anxiety symptoms. People who never returned home reported higher odds of depression (OR, 2.43; 95% CI, 1.70-3.49) and anxiety (OR, 3.04; 95% CI, 2.10-4.39) symptoms.

**Table 3.** Multivariable Analyses of Depression and Anxiety Symptoms Associated With Displacement

Characteristic^a^	Odds ratio (95% CI)	
	Depression symptoms (n = 40 056 618)	Anxiety symptoms (n = 51 473 559)
Displacement		
No displacement	1 [Reference]	1 [Reference]
<1 wk	1.08 (0.91-1.29)	1.13 (0.92-1.38)
>1 wk but <1 mo	1.77 (1.40-2.25)	1.54 (1.22-1.94)
1-6 mo	1.33 (0.95-1.87)	1.59 (1.15-2.20)
>6 mo	1.75 (1.04-2.96)	1.39 (0.82-2.33)
Never returned home	2.43 (1.70-3.49)	3.04 (2.10-4.39)
Age group, y		
18-25	1 [Reference]	1 [Reference]
26-34	1.08 (1.01-1.16)	1.04 (0.98-1.12)
35-49	0.87 (0.81-0.94)	0.88 (0.82-0.94)
50-64	0.59 (0.54-0.63)	0.57 (0.53-0.61)
65-74	0.33 (0.31-.036)	0.30 (0.28-0.32)
≥75	0.23 (0.21-0.25)	0.21 (0.19-0.23)
Sex at birth		
Female	0.96 (0.93-0.99)	1.31 (1.28-1.35)
Male	1 [Reference]	1 [Reference]
Sexual orientation		
Straight	1 [Reference]	1 [Reference]
Gay, lesbian, or bisexual	1.64 (1.58-1.71)	1.74 (1.67-1.81)
Race and ethnicity^b^		
Hispanic	0.85 (0.81-0.89)	0.78 (0.75-0.82)
Non-Hispanic Asian	0.81 (0.75-0.87)	0.61 (0.57-0.66)
Non-Hispanic Black	0.84 (0.80-0.88)	0.72 (0.69-0.75)
Non-Hispanic White	1 [Reference]	1 [Reference]
Non-Hispanic other^c^	1.07 (1.00-1.13)	1.04 (0.98-1.09)
Educational level		
≤High school diploma	1 [Reference]	1 [Reference]
Some college or associate’s degree	1.02 (0.98-1.05)	1.13 (1.09-1.16)
≥Bachelor’s or graduate degree	0.71 (0.68-0.73)	0.89 (0.86-0.92)
Annual income, $		
<35 000	1 [Reference]	1 [Reference]
35 000-74 999	0.82 (0.79-0.85)	0.83 (0.80-0.86)
75 000-149 999	0.62 (0.60-0.65)	0.65 (0.62-0.67)
≥150 000	0.38 (0.36-0.40)	0.43 (0.41-0.45)
Marital status		
Now married	1 [Reference]	1 [Reference]
Widowed, divorced, or separated	1.34 (1.29-1.39)	1.20 (1.16-1.24)
Never married	1.22 (1.17-1.27)	1.07 (1.03-1.11)
Household size, persons		
1-2	1 [Reference]	1 [Reference]
3-4	1.15 (1.11-1.19)	1.15 (1.11-1.19)
≥5	1.35 (1.27-1.44)	1.27 (1.20-1.35)
No. of children in the home		
No children	1 [Reference]	1 [Reference]
1-2	0.82 (0.79-0.86)	0.88 (0.85-0.92)
≥3	0.70 (0.65-0.76)	0.83 (0.77-0.89)
Homeownership		
Owner	1 [Reference]	1 [Reference]
Renter	1.19 (1.16-1.24)	1.23 (1.19-1.27)
Functional disability, severe		
No disability	1 [Reference]	1 [Reference]
1	3.40 (3.28-3.53)	3.14 (3.03-3.25)
≥2	8.10 (7.62-8.60)	6.92 (6.51-7.37)
Census region		
Northeast	1 [Reference]	1 [Reference]
South	1.08 (1.03-1.13)	1.04 (1.00-1.08)
Midwest	0.95 (0.91-1.00)	0.93 (0.90-0.97)
West	1.01 (0.96-1.06)	1.00 (0.96-1.04)

^a^ All variables listed are included in the multivariable analyses.

^b^ Self-reported by participants.

^c^ Includes American Indian or Alaska Native, Chamorro, Native Hawaiian, Other Pacific Islander, Samoan, and multiracial.

Females at birth had lower odds for depression symptoms (OR, 0.96; 95% CI, 0.93-0.99) but had higher odds for anxiety symptoms (OR, 1.31; 95% CI, 1.28-1.35) compared with males at birth. Other sociodemographic characteristics associated with higher odds of mental health symptoms were gay, lesbian, or bisexual sexual orientation; nonmarried status (widowed, divorced, separated, or never married); larger households; being a home renter, having functional disabilities; and living in the South (Table 3). For example, having 1 severe disability was associated with higher odds of having depression (OR, 3.40; 95% CI, 3.28-3.53) and anxiety (OR, 3.14; 95% CI, 3.03-3.25) symptoms, compared with those without any disability. Having children in the house (eg, ≥3) was associated with lower odds of depression (OR, 0.70; 95% CI, 0.65-0.76) and anxiety (OR, 0.83; 95% CI, 0.77-0.89) symptoms, compared with no children in the house. Racial and ethnic minority groups (eg, Asian) also had lower odds of depression (OR, 0.81; 95% CI, 0.75-0.87) and anxiety (OR, 0.61; 95% CI, 0.57-0.66) symptoms, compared with the White population. Similarly, those with higher income (eg, $75 000 to $149 999) had lower odds of depression (OR, 0.62; 95% CI, 0.60-0.65) and anxiety (OR, 0.65; 95% CI, 0.62-0.67) symptoms than those with income lower than $35 000.

## Discussion

In this cross-sectional study, natural disaster–induced displacement was associated with depression and anxiety symptoms. The longer the duration of displacement, the higher the odds of mental health symptoms in a pattern indicating a dose-response association. Our findings, based on nationally representative data, are generalizable to the US adult population. We examined various types of natural disasters that extended beyond those in previous studies, which had more modest sample sizes and focused on individual disaster events in the US.

Our finding that an association exists between displacement duration and mental health outcomes is important. Only a limited number of studies have examined longer duration of displacements or individuals who never returned home after displacement. This literature includes a study of New Orleans residents after Hurricane Katrina, which found a higher prevalence of mild to severe mental illnesses among those who remained displaced from the city after 1 year compared with those who returned (51% vs 31%).^10^

Furthermore, another study examined 4 dimensions of displacement: distance from the predisaster community, type of postdisaster housing, number of postdisaster moves, and time spent in temporary housing.^3^ That study, which focused on New Orleans residents displaced by Hurricane Katrina, found that unstable postdisaster housing experiences and relocation (not returning home) were associated with more adverse mental health outcomes.^3^ The current study illustrated that the duration of displacement may be an additional key aspect of the displacement profile that should be considered when studying the association between displacement and mental health outcomes.

In a previous study,^20^ experiencing fires and multiple disasters in the past year was associated with a longer duration of displacement (ie, displaced for more than 6 months or never returned home). This finding suggests that, for populations displaced by fires or multiple disasters in a year, mental health services will be more crucial. Furthermore, whereas setting up mental health services in a disaster area can target people who return, mental health practitioners will need to develop care models to reach permanently displaced individuals.

We cannot be certain whether experiencing displacement was the sole factor in depression and anxiety symptoms or whether experiencing other outcomes associated with disasters, such as fear for one’s life during the disaster and property damage, also played a role in mental health condition. A 1-year follow-up study of New Orleans residents who were displaced and who did not return home after Hurricane Katrina found that severe damage and destruction of their homes was a major factor in mental illness.^10^

Our finding that racial and ethnic minority groups (Asian, Black, and Hispanic) had lower odds of experiencing depression and anxiety symptoms compared with White individuals is interesting. Although studies found that racial and ethnic minority people were more likely to experience mental health symptoms compared with their White counterparts after natural disasters,^23,24^ a systematic review found that, after adjusting for socioeconomic status and other social support, race was no longer a factor in some studies.^6^

We found the highest odds of mental health symptoms among participants who reported functional disabilities than any other characteristics examined. Studies on the mental health of people with disabilities after natural disasters are still limited. A study of Hurricane Harvey in Texas found that health care access was limited for those with disabilities^23^ and may have a causal association with higher rates of anxiety and depression among this population. We recommend that future studies focus on people with disabilities and on mental health services in the context of natural disasters for this socially vulnerable population. The availability of mental health resources in both the short and long term will be critical. The mental health outcomes of displacement can continue well after the disaster occurs, as individuals struggle with the ambiguity about not only their future but also the process of creating what that future entails. This emotional distress can be especially difficult for underresourced or underrepresented groups, including children (aged <18 years) and older adults (aged ≥65 years), but also for individuals with existing stressors, such as chronic physical and mental health issues.^25,26,27^ During a disaster and the weeks and months afterward, the inability to access mental health professionals and resources can intensify adverse health outcomes.^28,29^ This would require that health care practitioners are appropriately trained to recognize and provide tailored care to patients with a history of disaster-induced displacement. Training and educational programs for health care practitioners can benefit from the inclusion of various stakeholders, including communities at risk of natural disasters, mental health care practitioners, and people who have experienced displacement.

### Limitations

We note several limitations of this study. First, the HPS is associated with low response rates; the survey used in this study had a mean response rate of 6.6%. The issue of low response rates of the HPS has been described elsewhere,^20^ and the Census Bureau applies nonresponse weighting adjustments to minimize nonresponse bias.^30^ Second, the HPS relied on self-reported measures, including exposure (displacement and duration of displacement) and outcomes (depression and anxiety symptoms). The HPS mental health questions relied on validated PHQ-2 and GAD-2 measures. Finally, the cross-sectional nature of the data precludes any conclusion about causality as we could not be sure if people who reported depression and anxiety symptoms before displacement were more likely to experience the symptoms after displacement. It is possible that people with preexisting mental health symptoms prior to the disaster choose not to return to their predisaster residences or not to return after a longer delay. This kind of methodological limitation is common in the current literature on displacement and mental health, prompting the need for prospective and longitudinal studies that consider baseline predisaster mental health characteristics.^6,31^

## Conclusions

In this cross-sectional study, disaster-induced displacement was associated with depression and anxiety symptoms with higher risks among individuals who were displaced over a longer duration or who never returned home. As climate change increases the severity and frequency of many of the natural disasters we examined, mental health services for a growing number of displaced populations will become more critical.

## Notes

Supplement 1.
eMethods
**eTable.** Unweighted Missing Data Among Those Who Responded to Displacement Question

Supplement 2.
Data Sharing Statement

## References

[1] Saeed SA, Gargano SP. Natural disasters and mental health. Int Rev Psychiatry. 2022;34(1):16-25. doi:10.1080/09540261.2022.2037524 35584023

[2] Munro A, Kovats RS, Rubin GJ, Waite TD, Bone A, Armstrong B; English National Study of Flooding and Health Study Group. Effect of evacuation and displacement on the association between flooding and mental health outcomes: a cross-sectional analysis of UK survey data. Lancet Planet Health. 2017;1(4):e134-e141. doi:10.1016/S2542-5196(17)30047-5 28944321 PMC5597543

[3] Fussell E, Lowe SR. The impact of housing displacement on the mental health of low-income parents after Hurricane Katrina. Soc Sci Med. 2014;113:137-144. doi:10.1016/j.socscimed.2014.05.025 24866205 PMC4096953

[4] Pfefferbaum B, Nitiéma P, Newman E. A meta-analysis of intervention effects on depression and/or anxiety in youth exposed to political violence or natural disasters. Child Youth Care Forum. 2019;48(4):449-477. doi:10.1007/s10566-019-09494-9

[5] Kar N, Bastia BK. Post-traumatic stress disorder, depression and generalised anxiety disorder in adolescents after a natural disaster: a study of comorbidity. Clin Pract Epidemiol Ment Health. 2006;2:17. doi:10.1186/1745-0179-2-17 16869979 PMC1563462

[6] Chen S, Bagrodia R, Pfeffer CC, Meli L, Bonanno GA. Anxiety and resilience in the face of natural disasters associated with climate change: a review and methodological critique. J Anxiety Disord. 2020;76:102297. doi:10.1016/j.janxdis.2020.102297 32957002

[7] Kaya Kılıç A, Uğur SB, Bademli K. The relationship between post-traumatic stress, hopelessness and resources adequacy in fire disaster survivors: a mediation analysis. Clin Soc Work J. Published online July 22, 2024. doi:10.1007/s10615-024-00956-9

[8] Labarda CE, Jopson QDQ, Hui VKY, Chan CS. Long-term displacement associated with health and stress among survivors of Typhoon Haiyan. Psychol Trauma. 2020;12(7):765-773. doi:10.1037/tra0000573 32212778

[9] Rhodes J, Chan C, Paxson C, Rouse CE, Waters M, Fussell E. The impact of Hurricane Katrina on the mental and physical health of low-income parents in New Orleans. Am J Orthopsychiatry. 2010;80(2):237-247. doi:10.1111/j.1939-0025.2010.01027.x 20553517 PMC3276074

[10] Sastry N, VanLandingham M. One year later: mental illness prevalence and disparities among New Orleans residents displaced by Hurricane Katrina. Am J Public Health. 2009;99(suppl 3):S725-S731. doi:10.2105/AJPH.2009.174854 19890178 PMC2774198

[11] Wadsworth ME, Santiago CD, Einhorn L. Coping with displacement from Hurricane Katrina: predictors of one-year post-traumatic stress and depression symptom trajectories. Anxiety Stress Coping. 2009;22(4):413-432. doi:10.1080/10615800902855781 19343597

[12] Centers for Disease Control and Prevention (CDC). Assessment of health-related needs after Hurricanes Katrina and Rita–Orleans and Jefferson Parishes, New Orleans area, Louisiana, October 17-22, 2005. MMWR Morb Mortal Wkly Rep. 2006;55(2):38-41. http://dx.doi.org/. 16424857

[13] Schwartz RM, Gillezeau CN, Liu B, Lieberman-Cribbin W, Taioli E. Longitudinal impact of Hurricane Sandy exposure on mental health symptoms. Int J Environ Res Public Health. 2017;14(9):957. doi:10.3390/ijerph14090957 28837111 PMC5615494

[14] Crimmins AR, Avery CW, Easterling DR, Kunkel KE, Stewart BC, Maycock TK, eds. Fifth National Climate Assessment. US Global Change Research Program; 2023. doi:10.7930/NCA5.2023.RiB

[15] Equator Network. STROBE statement: checklist of items that should be included in reports of cross-sectional studies. Accessed July 28, 2022. https://www.equator-network.org/wp-content/uploads/2015/10/STROBE_checklist_v4_cross-sectional.pdf

[16] U.S. Census Bureau. Source of the data and accuracy of the estimates for the Household Pulse Survey. Household Pulse Survey technical documentation. Accessed November 16, 2023. https://www.census.gov/programs-surveys/household-pulse-survey/technical-documentation/source-accuracy.2023.html#list-tab-1670696597

[17] National Center for Health Statistics; U.S. Census Bureau. Household Pulse Survey, 2020–2024. Anxiety and depression. Accessed October 15, 2024. https://www.cdc.gov/nchs/covid19/pulse/mental-health.htm

[18] Kroenke K, Spitzer RL, Williams JBW. The Patient Health Questionnaire-2: validity of a two-item depression screener. Med Care. 2003;41(11):1284-1292. doi:10.1097/01.MLR.0000093487.78664.3C 14583691

[19] Kroenke K, Spitzer RL, Williams JBW, Monahan PO, Löwe B. Anxiety disorders in primary care: prevalence, impairment, comorbidity, and detection. Ann Intern Med. 2007;146(5):317-325. doi:10.7326/0003-4819-146-5-200703060-00004 17339617

[20] Aung TW, Sehgal AR. Prevalence, correlates, and impacts of displacement because of natural disasters in the United States from 2022 to 2023. Am J Public Health. 2025;115(1):55-65. doi:10.2105/AJPH.2024.307854 39447105 PMC11628721

[21] Jensen E, Jones N, Orozco K, . Measuring racial and ethnic diversity for the 2020 Census. Accessed January 8, 2025. https://www.census.gov/newsroom/blogs/random-samplings/2021/08/measuring-racial-ethnic-diversity-2020-census.html.

[22] Lumley T, Scott A. Fitting regression models to survey data. Stat Sci. 2017;32(2):265-278. doi:10.1214/16-STS605

[23] Flores AB, Collins TW, Grineski SE, Chakraborty J. Disparities in health effects and access to health care among Houston area residents after Hurricane Harvey. Public Health Rep. 2020;135(4):511-523. doi:10.1177/0033354920930133 32539542 PMC7383766

[24] Fitzpatrick KM. Post-traumatic stress symptomatology and displacement among Hurricane Harvey survivors. Soc Sci Med. 2021;270:113634. doi:10.1016/j.socscimed.2020.113634 33433371

[25] Patel SS, Robb K, Pluff C, Maldonado E, Tatar G, Williams T. Elevating mental health disparities and building psychosocial resilience among BIPOC children and youth to broaden the climate and health discourse. J Appl Res Child. 2021;12(1). doi:10.58464/2155-5834.1457

[26] Davis CR, Berke P, Holloman DE, . Support strategies for socially marginalized neighborhoods likely impacted by natural hazards. July 2021. Accessed December 31, 2024. https://coastalresiliencecenter.unc.edu/wp-content/uploads/sites/845/2021/07/Support-Strategies-for-Socially-Marginalized-Neighborhoods.pdf

[27] Rafiey H, Momtaz YA, Alipour F, . Are older people more vulnerable to long-term impacts of disasters? Clin Interv Aging. 2016;11:1791-1795. doi:10.2147/CIA.S122122 27994445 PMC5153288

[28] North CS, Pfefferbaum B. Mental health response to community disasters: a systematic review. JAMA. 2013;310(5):507-518. doi:10.1001/jama.2013.107799 23925621

[29] Schwartz RM, Liu B, Lieberman-Cribbin W, Taioli E. Displacement and mental health after natural disasters. Lancet Planet Health. 2017;1(8):e314. doi:10.1016/S2542-5196(17)30138-9 29628166

[30] Peterson S, Toribio N, Farber J, Hornick D. Nonresponse bias report for the 2020 Household Pulse Survey. US Census Bureau. March 24, 2021. Accessed November 16, 2023. https://www2.census.gov/programs-surveys/demo/technical-documentation/hhp/2020_HPS_NR_Bias_Report-final.pdf

[31] Goldmann E, Galea S. Mental health consequences of disasters. Annu Rev Public Health. 2014;35:169-183. doi:10.1146/annurev-publhealth-032013-182435 24159920

