# Supplementary material for: Depression and Anxiety Symptoms in Adults Displaced by Natural Disasters
Source: JAMA Netw Open. 2025 Aug 22;8(8):e2528546. doi: 10.1001/jamanetworkopen.2025.28546 (PMC12374215; doi:10.1001/jamanetworkopen.2025.28546)
Supplement: Supplement 1. — eMethods eTable. Unweighted Missing Data Among Those Who Responded to Displacement Question [file jamanetwopen-e2528546-s001.pdf]

Supplemental Online Content

Aung TW, O'Donnell K, De Luca S, Gunzler D. Depression and anxiety symptoms in adults displaced by natural disasters. *JAMA Netw Open*. 2025;8(8):e2528546.
doi:10.1001/jamanetworkopen.2025.28546

eMethods

eTable. Unweighted Missing Data Among Those Who Responded to Displacement Question

This supplemental material has been provided by the authors to give readers additional information about their work.

© 2025 Aung TW et al. *JAMA Network Open*

eMethods

Study sample

A total of 826,941 respondents participated in the Household Pulse Survey between December 2022 and October 2023. Of those, 727,001 responded to the questionnaire on, “In the past year, were you displaced from your home because of a natural disaster?” and were eligible for the study. The eTable illustrates the number and proportion of missing data among the participants eligible for our study. Missing data were dropped resulting in the final sample size of 650,632 participants in our analyses.

eTable: Unweighted missing data among those who responded to displacement question

Characteristics	n (%)
**Dependent variable**	
Depressive symptoms	14,103 (1.94%)
Anxiety symptoms	13,611 (1.87%)
**Independent variable**	
Age, years	0 (0%)
Sex at birth	0 (0%)
Sexual orientation	7,630 (1.05%)
Race & ethnicity	0 (0%)
Education	0 (0%)
Annual income	56,056 (7.71%)
Marital status	2,456 (0.34%)
Household size	0 (0%)
Number of children in home	0 (0%)
Housing type	39,200 (5.39%)
Home ownership	32,947 (4.53%)
Disability	22,883 (3.15%)
Census region	0 (0%)

© 2025 Aung TW et al. *JAMA Network Open*
