# Supplementary material for: Depression and Anxiety Symptoms in Adults Displaced by Natural Disasters
Source: JAMA Netw Open. 2025 Aug 22;8(8):e2528546. doi: 10.1001/jamanetworkopen.2025.28546 (PMC12374215; doi:10.1001/jamanetworkopen.2025.28546)
Supplement: Supplement 2. — Data Sharing Statement [file jamanetwopen-e2528546-s002.pdf]

Data Sharing Statement

Aung. Depression and Anxiety Symptoms in Adults Displaced by Natural Disasters. *JAMA Netw Open*. Published August 22, 2025. doi:10.1001/jamanetworkopen.2025.28546

Data
Data available: Yes
Data types: Deidentified participant data
How to access data: https://www.census.gov/programs-surveys/household-pulse-survey/data/datasets.2023.html#list-tab-1264157801
When available: With publication

Supporting Documents
Document types: None

Additional Information
Who can access the data: The data is publicly available on the Census website.
Types of analyses: For any purpose
Mechanisms of data availability: The data is publicly available on the Census website.
